# Laser-Generated Guided Waves for Damage Detection in Metal-Lined Composite-Overwrapped Pressure Vessels

**DOI:** 10.3390/polym14183823

**Published:** 2022-09-13

**Authors:** Jinling Zhao, Lehui Yang, Hongyuan Wang, Jianping Zhao, Nian Li, Le Chang, Hongli Ji, Jinhao Qiu

**Affiliations:** 1 School of Mechanical and Power Engineering, Nanjing Tech University, Nanjing 211816, China; 2 Jiangsu Key Lab of Design and Manufacture of Extreme Pressure Equipment, Nanjing Tech University, Nanjing 211816, China; 3 State Key Laboratory of Mechanics and Control of Mechanical Structures, Nanjing University of Aeronautics and Astronautics, Nanjing 210016, China

**Keywords:** composite-overwrapped pressure vessel, non-destructive evaluation, laser-ultrasonics, local wavenumber estimation

## Abstract

This paper characterizes laser-generated guided waves in a metal-lined composite-overwrapped pressure vessel (COPV) to assess typical damage, including interfacial debonding and low-velocity impact damage. First, an eigenfrequency approach that avoids additional coding is utilized to theoretically analyze the dispersion characteristics of a COPV. The theoretical results show that interfacial debonding significantly alters dispersion curves, and the wavenumber of the L(0, 1) mode is sensitive to impact damage. Experimental verifications were conducted based on the full wavefield acquired using a scanning laser-ultrasonic system with a repetition rate of 1 kHz. By comparing the experimental dispersion curves with the theoretical ones, it was found that the metal-composite interface was not bonded. In addition, a local wavenumber estimation method was established to detect the impact damage by obtaining the spatial distribution of the wavenumber of the L(0, 1) mode.

## 1. Introduction

**Figure 1:**
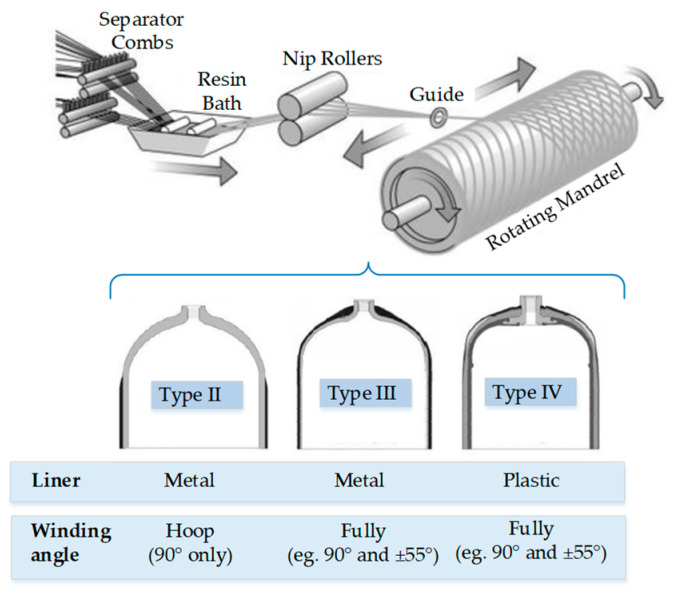
Illustration of the filament winding process and typical COPVs.

Composite-overwrapped pressure vessels, which reduce weight while improving structural performance, have been increasingly used in aerospace, automotive and other industrial fields [1]. Three types of COPVs are commonly used in the hydrogen storage field. A type II COPV has a thick metallic-liner hoop wrapped with carbon-fiber-reinforced polymers (CFRPs) on the cylindrical part, whereas type III and IV COPVs are fully wrapped with CFRPs with metal and plastic liners, respectively [2,3]. An illustration of the filament winding process and sketches of Type II, III and IV COPVs are provided in Figure 1.

Type III COPVs are usually used for long-term hydrogen storage [4]. According to the failure mechanisms of type III COPVs [5,6,7,8,9], when subjected to working loads, debonding at the interface that connects the liner and the outer layer, as well as low-velocity impact damage, may occur, endangering the structure’s integrity and even human safety. To assess these damages, non-destructive evaluations [10,11,12] are required.

Ultrasonic-guided waves (UGWs) are promising for damage detection in COPVs due to their long propagation distances in hollow-cylindrical waveguides and high sensitivities to various damage [13]. 

There are three types of UGWs in cylinders, including the axisymmetric longitudinal mode L(0, *n*), axisymmetric torsional mode T(0, *n*), and non-axisymmetric flexural mode F(*m*, *n*) [14]. The transfer-matrix method [15] and the global-matrix method [16] are commonly used to analyze the wave characteristics of multi-layer structures, whereas the semi-analytical finite element (SAFE) method provides more efficient and robust results [17]. Hakoda et al. [18] recently combined the Floquet periodic boundary conditions with the eigenfrequency method to analyze the wave characteristics of isotropic pipes efficiently. 

Regarding the experiments, the scanning laser-ultrasonic method [19,20] has been widely used to evaluate plate structures and simple pipes [21,22,23,24,25,26] due to its numerous advantages, such as its non-contact nature, remote excitation, high repetition rate and high resolution. Dispersion curves can be easily obtained because the scanning laser-ultrasonic method can acquire adequate spatial ultrasonic information [27]. Furthermore, local wavenumber features can be extracted from the full wavefield and have been used to evaluate the wall thinning of metallic pipes and the impact delaminations of complex composites [23]. In comparison to wave energy [21], wavenumber features are associated with more damage information, such as damage depth, etc. Segers et al. [24] proposed an adaptive filtering method to improve the local wavenumber method and visualize different damage shapes and depths, which was similar to the work of Tian et al. [25]. The use of laser ultrasonics on a metal-composite plate structure to visualize impact damage was recently investigated [26].

However, to the best of our knowledge, there are few reports on scanning laser-ultrasonics in Type III COPVs. Type III COPVs, in contrast to simple-plate or pipe waveguides, are multi-layered metal and CFRPs materials with hollow cylindrical geometries, making the guided wave properties more complex. Furthermore, the mechanism of interaction between the guided waves and structural damage (the interfacial debonding and impact damage) in Type III COPVs is unknown, rendering the selection of the wave characteristics and the design of detection schemes difficult. As a result, a thorough theoretical investigation of wave characteristics in type III COPVs is required.

**Figure 2:**
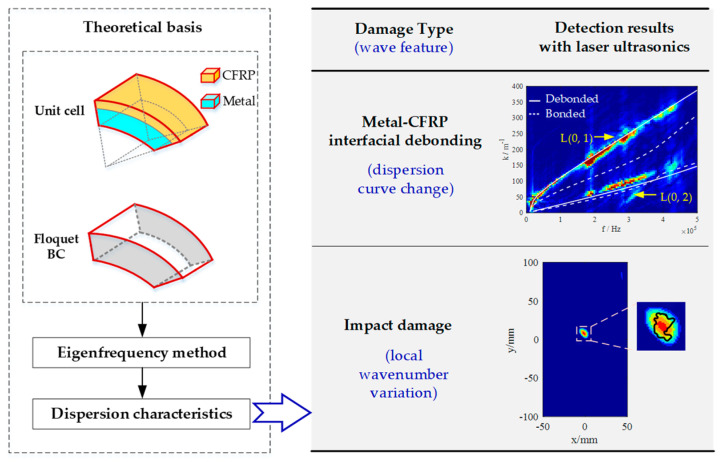
The overall framework and main results of the paper.

The purpose of this paper is to investigate laser-based UGWs for the detection of typical damage in metal-lined COPVs. It first builds a theoretical model of guided waves in metal-lined COPVs, then investigates wave-damage interactions to select wave features, and finally employs the laser-ultrasonic method for damage evaluation. Figure 2 shows the overall framework and main results of the paper.

The remainder of this paper is structured as follows. Section 2 builds a theoretical model based on the eigenfrequency method to analyze the wave dispersion characteristics of metal-lined COPVs. To represent an infinite COPV, a specific unit cell with Floquet boundary conditions is constructed. Section 3 investigates the effects of damage on the wave characteristics. Section 4 describes the local wavenumber estimation method. Section 5 uses a scanning laser-ultrasonic system to investigate the guided waves in a COPV. The interface debonding was determined by comparing the dispersion characteristics calculated theoretically and those measured experimentally. Meanwhile, the impact damage was successfully identified using the local wavenumber estimation method. Conclusions are drawn in Section 6.

## 2. Theoretical Background 

To investigate the dispersion characteristics of metal-lined COPVs, the eigenfrequency method acting on a specific unit cell was utilized. First, it was demonstrated that representing the entire waveguide with a unit cell is feasible. By imposing the appropriate boundary conditions on the unit cell, the eigenfrequencies of different wavenumbers could then be efficiently calculated in commercial finite element software, yielding the dispersion characteristics of the metal-lined COPVs.

### 2.1. Unit Cell Representation

**Figure 3:**
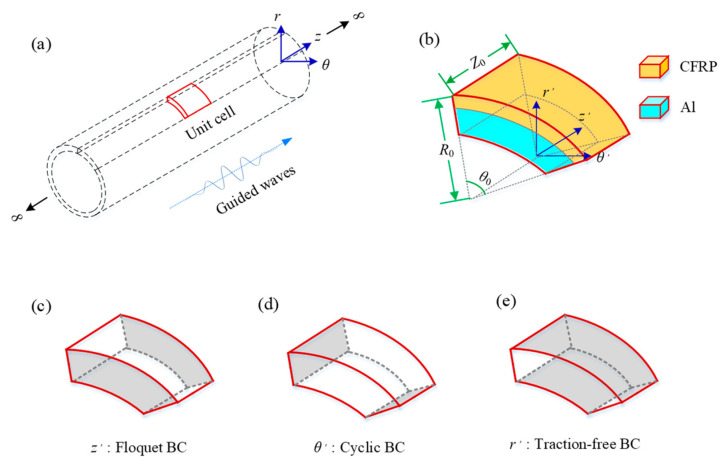
Illustration of (**a**) an infinite COPV waveguide, (**b**) a unit cell (**c**–**e**) the boundary conditions along $z{}^{\prime }$, $\theta {}^{\prime }$ and $r{}^{\prime }$ in the unit cell.

The infinite COPV waveguide and the unit cell are illustrated in Figure 3a,b, respectively. The filament-wound layers were considered to be multiple layers stacked sequentially, which is reasonable at relatively low frequencies where the guided wave structures are simple.

The wavefield in the infinite COPV was assumed as

(1) $${u(r,\theta ,z,t)=U(r)e}^{{-i(m\theta +k}_{z}z)+i\omega t}$$

where *ω* is the angular frequency, $k_{z}$ is the angular wavenumber along the axial direction and *m* is the circumferential order with

(2) $${m=k}_{\theta }R_{0}$$

Here, $k_{\theta }$ is the angular wavenumber along the circumferential direction. 

To demonstrate the feasibility of representing the entire waveguide with a unit cell is to prove the equivalence of the dispersion relation and the wave structure between the entire waveguide and the unit cell.

In the reciprocal-lattice space [28], the variation of the geometric variables between the infinite pipe and the unit cell was

(3) $$r=r{}^{\prime }$$

(4) $${\theta =\theta {}^{\prime }+m}_{\theta }\theta_{0}$$

(5) $${z=z{}^{\prime }+m}_{z}Z_{0}$$

The changes in wavenumbers were

(6) $$k_{r}=0$$

(7) $${m=k}_{\theta }R_{0}{=k}_{\theta }^{{}^{\prime }}R_{0}{+n}_{\theta }\frac{2\pi }{\theta_{0}}$$

(8) $$k_{z}{=k}_{z}^{{}^{\prime }}{+n}_{z}\frac{2\pi }{z_{0}}$$

where $m_{\theta },m_{z},n_{\theta },n_{z}$ are integer values [28]. Wavenumbers $k_{\theta }^{{}^{\prime }}$ and $k_{z}^{{}^{\prime }}$ exist in the intervals

(9) $$-\frac{\pi }{\theta_{0}R_{0}}\leq k_{\theta }^{{}^{\prime }}\leq \frac{\pi }{\theta_{0}R_{0}}$$

(10) $$-\frac{\pi }{Z_{0}}\leq k_{z}^{{}^{\prime }}\leq \frac{\pi }{Z_{0}}$$

Equations (9) and (10) are referred to as the first Brillouin Zone [28], which is the focus zone of the eigenfrequency approach because when the periodicity is simple, the dispersion curves are the same for the other regions [18]. 

By substituting Equations (3)–(8) into Equation (1) and simplifying the terms, we obtain

(11) $${u(r,\theta ,z,t)=e}^{{-i(k}_{\theta }^{{}^{\prime }}m_{\theta }R_{0}\theta_{0}{+k}_{z}^{{}^{\prime }}m_{z}Z_{0})}{U(r}^{{}^{\prime }}{)e}^{{-i(m\theta }^{{}^{\prime }}{+k}_{z}z^{{}^{\prime }})+i\omega t}$$

If $e^{{-i(k}_{\theta }^{{}^{\prime }}m_{\theta }R_{0}\theta_{0}{+k}_{z}^{{}^{\prime }}m_{z}Z_{0})}$ is a constant, there is

(12) $${u(r,\theta ,z,t)=CU(r}^{{}^{\prime }}{)e}^{{-i(m\theta }^{{}^{\prime }}{+k}_{z}z^{{}^{\prime }})+i\omega t}$$

Comparing Equation (12) with Equation (1), it is observed that the wavenumber, frequency and wave structures of the infinite pipe solution are the same as those from the unit cell, demonstrating the feasibility of representing the whole pipe using the unit cell.

### 2.2. Eigenfrequency Approach

In addition to the geometric assumptions of the unit cell, specific boundary conditions are necessary, including the Floquet BC along the axial direction [18,29], cyclic BC along the circumferential direction, and of course, the traction-free BC, as shown in Figure 3c–e, respectively.

The Floquet BC, defined as

(13) $$u_{d}{=u}_{s}e^{{-ik}_{F}{(z}_{d}{-z}_{s})}$$

is applied to two parallel surfaces along the axial direction to set the wavenumber constraint between the two positions. The subscripts *d* and *s* denote the destination plane and the starting plane, respectively. $k_{F}$ is the angular wavenumber applied in the cell along the axial direction.

Recalling the wavefield equation in the cell and substituting Equation (12) into Equation (13), we obtain

(14) $${CU(r}^{{}^{\prime }}{)e}^{{-i(m\theta }^{{}^{\prime }}{+k}_{z}z^{{}^{\prime }})+i\omega t}{丨}_{z^{{}^{\prime }}=0}{=CU(r}^{{}^{\prime }}{)e}^{{-i(m\theta }^{{}^{\prime }}{+k}_{z}z^{{}^{\prime }})+i\omega t}{丨}_{z^{{}^{\prime }}{=-Z}_{0}}e^{{-ik}_{F}Z_{0}}$$

Then, we obtain

(15) $$e^{{-ik}_{z}Z_{0}}{=e}^{{-ik}_{F}Z_{0}}$$

which indicates the feasibility of using the wavenumber $k_{F}$ in the Floquet BC to represent the wavenumber $k_{z}$ in the infinite pipe. 

For waves propagating in the circumferential direction, the cyclic BC can be imposed on the unit cell, which has similar properties to the Floquet BC, namely

(16) $$u_{d}{=T(\theta }_{0}{)u}_{s}e^{{-ik}_{\theta }R_{0}\theta_{0}}$$

where $u_{d}$ and $u_{s}$ represent the displacements in the default Cartesian coordinates. *T* is a rotation matrix for transferring the wavefield of the source plane to the destination plane. 

In this paper, the focus was on the waves propagating along the axial direction, so the circumferential wavenumber was assumed to be zero. In this case, the continuity BC can be used instead, i.e.,

(17) $$u_{d}{=T(\theta }_{0}{)u}_{s}e^{{-ik}_{\theta }R_{0}\theta_{0}}|{\;}_{\theta_{0}\to \;0}\approx u_{s}$$

where the rotation matrix is approximately equal to the unit matrix when $\theta_{0}$ takes a small value. 

For a given wavenumber $k_{F}$, the eigenfrequencies can be easily obtained with commercial finite element software. With this method of eigenfrequency, a parametric study of $k_{F}$ in the first Brillouin zone was carried out to derive the dispersion relation of ${(\omega ,k}_{z})$. The corresponding eigenvectors were exactly the same as the wave structure, *U*(*r*). 

## 3. Wave Features of COPVs with Damages

In this section, the dispersion curves of guided waves in metal-lined COPV are calculated using the eigenfrequeny approach. The effects of the interfacial debonding and impact damage on the wave characteristics are analyzed.

The inner and outer diameters of the aluminum liner were 176 mm and 180 mm, respectively. The filament winding angles were of the order [90_2_/(±55)_3_/90_2_], and the thickness of the single layer was 0.2 mm. The elastic constants and densities of the aluminum- and carbon-fiber-reinforced polymers are given in Table 1.

The eigenfrequency approach was implemented in COMSOL Multiphysics [29], a general finite element software that can solve the eigenfrequency problem, impose a Floquet BC and cyclic BC and conduct the parametric study easily.

In the eigenfrequency approach, $\theta_{0}\;$ and $Z_{0}$ should be small enough to extend the first Brillouin Zone [18] according to Equations (9) and (10). In this paper, the unit cell was established as shown in Figure 3b with $\theta_{0}=0.6^{o}$ and $Z_{0}=0.4\mathrm{mm}$, where $Z_{0}$ equalled 1/10 of the pipe thickness. The unit cell was discretized with quadratic tetrahedron elements with an average size of 0.2 mm. The Floquet BC was imposed on two parallel surfaces along the wave propagation direction (axial), and a continuity BC was set on the circumferential faces to simulate the axial symmetric modes. A parametric study of the wavenumber, $k_{F}$, set in the Floquet BC was carried out with $k_{F}{=[0,\pi /Z}_{0}]$ at an interval of ${\pi /(100Z}_{0})$. The eigenfrequency under each $k_{F}$ was obtained with the finite element software to derive the frequency–wavenumber relation, i.e., the dispersion characteristics. 

**Figure 4:**
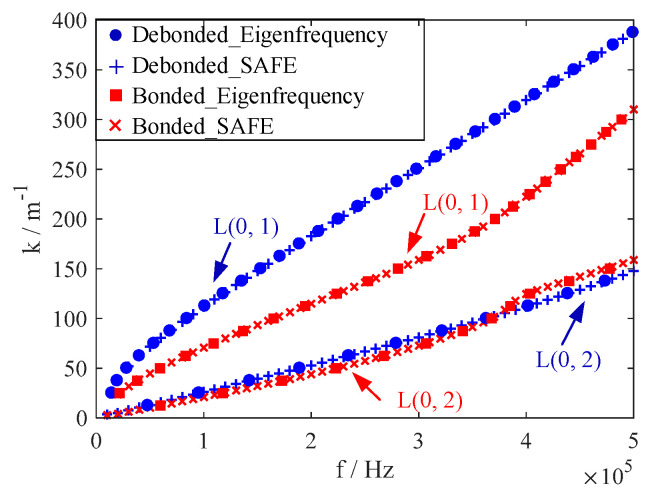
Dispersion curves in COPVs with bonded and debonded Al-CFRP interfaces.

The dispersion curves are drawn in Figure 4. Corresponding to the subsequent experiment in Section 5, only the fundamental UGW modes were considered here. The SAFE method [17] was also performed to validate the eigenfrequency approach. The cross-section of the pipe was discretized with one-dimensional quadratic elements with 10 elements for the CFRP layer and 10 elements for the Al layer. The results are plotted as red crosses in Figure 4, matching well with the results of the eigenfrequency approach.

In the extreme case where the metal and the composite are not bonded at all, the guided wave propagating in the outer layer cannot enter the metal liner, and vice versa. As shown in Figure 4, the dispersion curves were calculated in a debonded COPV where the guided wave propagated only in the CFRP layer. 

The results show that when the COPV is debonded, the dispersion curves vary significantly, indicating that the dispersion curve can be a good choice for detecting interfacial debonding damage. Furthermore, compared to the L(0, 2) mode, the dispersion curves of the L(0, 1) mode were more sensitive when interface debonding was present. More quantitative studies on the dispersion characteristics at various degrees of interfacial debonding states are required in the future.

**Figure 5:**
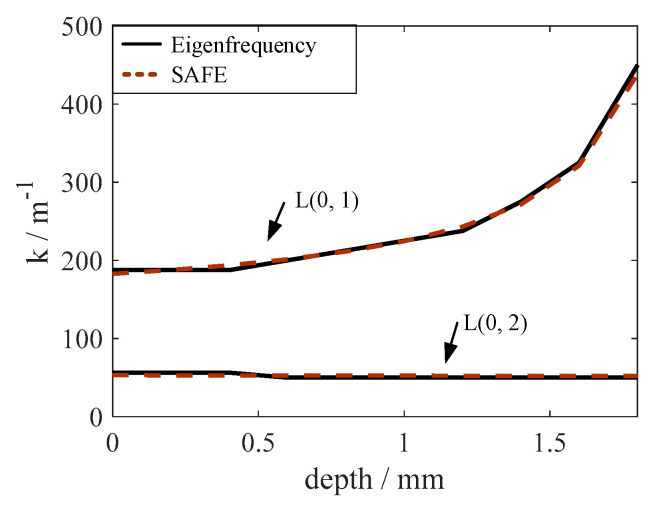
Wavenumber vs. depth of impact damage.

The damage produced by low-velocity impact testing and quasi-static indentation loading can be considered identical [30,31]. Therefore, the severity of the impact damage can be approximated with the indentation depth. Considering the different indentation depths in the composite layer, the corresponding wavenumber of 200 kHz was calculated, as shown in Figure 5. The wavenumber of the L(0, 1) mode was found to be suitable for impact damage detection because it varied greatly with the damage depth, while L(0, 2) was almost constant.

## 4. Local Wavenumber Estimation Method

According to the theoretical investigation, the wavenumber of the L(0, 1) mode is suitable for impact damage detection. This section describes the local wavenumber estimation method for impact damage imaging by evaluating the wavenumber of the L(0, 1) mode at each spatial point of interest. 

**Figure 6:**
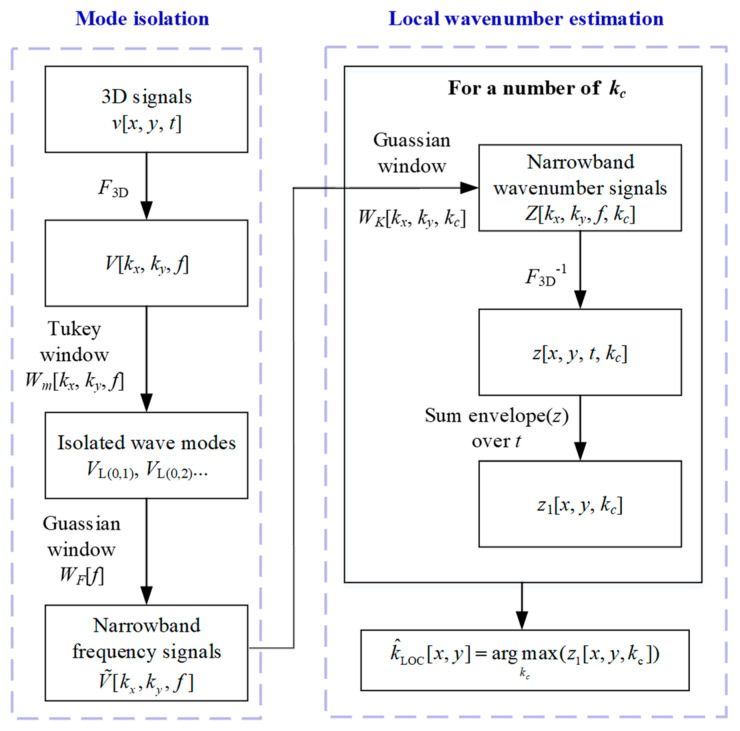
Flow diagram of the local wavenumber estimation.

Because the laser-ultrasonic wavefield contained multiple UGW modes, the mode isolation should have been performed before estimating the local wavenumber. Figure 6 shows the detailed procedure of the local wavenumber estimation method [23]. In this section, the default Cartesian system was used for convenience.

### 4.1. Wave Mode Isolation

Three-dimensional Fourier transformation is defined as follows:

(18) $$V\left(k_{x}{,k}_{y},f\right)=\int_{-\infty }^{\;\infty }\int_{-\infty }^{\infty }\int_{-\infty }^{\infty }{v(x,y,t)e}^{{-i(2\pi ft-2\pi k}_{x}{x-2\pi k}_{y}y)}dtdxdy$$

Equation (18) converts the ultrasonic signals from the time–space region to the frequency–wavenumber domain. Here, $k_{x}$ and $k_{y}$ are the wavenumber components along the *x* and *y* axes, respectively. v(x,y,t) is the original time–space wavefield data matrix, and ${V(k}_{x}{,k}_{y},f)$ is in the frequency–wavenumber domain. 

Different filter windows regarding the wavenumber or frequency can be applied independently to the frequency–wavenumber domain signals, thus achieving mode isolation. In this paper, a two-dimensional Tukey window was used to retain the wavenumber region of interest and eliminate the rest, while a one-dimensional Gaussian window was used to filter the frequencies.

**Figure 7:**
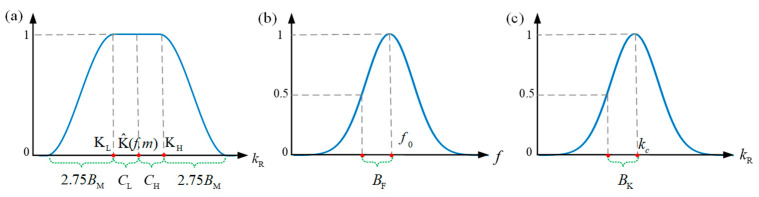
(**a**) Tukey window in the wavenumber $k_{R}$ domain, (**b**) Gaussian window in the frequency *f* domain, (**c**) Gaussian window in the wavenumber $k_{R}$ domain. Note that $k_{R}=\sqrt{k_{x}^{2}{+k}_{y}^{2}}$.

The Tukey window curve is shown in Figure 7a, where the horizontal axis represents the radial wavenumber according to

(19) $$k_{R}=\sqrt{k_{x}^{2}{+k}_{y}^{2}}$$

The Tukey window specifies an all-pass region $[{\hat{K}}_{L}(f,m),{\hat{K}}_{H}\left(f,m)\right]$ as follows:

(20) $$\begin{array}{c} K_{L}=\hat{K}\left(f,m\right){-C}_{L}\\ K_{H}=\hat{K}\left(f,m\right){+C}_{H} \end{array}$$

Here, $\hat{K}\left(f,m\right)$ is the central wavenumber of the target mode to be isolated. $C_{L}$ and $C_{H}$ are the lower and upper cutoffs, respectively. The Tukey window is a cosine-tapered window defined as

(21) $$W_{m}{[k}_{x}{,k}_{y},f]=\frac{1}{2}+\frac{1}{2}\cos\left(\frac{{\pi (k}_{R}{-K}_{B}\left(f,m)\right)}{2{.75B}_{M}}\right)$$

where $B_{M}$ is the half-power taper bandwidth, and

(22) $$K_{B}(f,m)=\{\begin{array}{c} K_{L}{\;K}_{L}-2{.75B}_{M}{<k}_{R}\leq K_{L}\;\\ k_{R}{\;K}_{L}{<k}_{R}\leq K_{H}\;\\ K_{H}{\;K}_{H}{<k}_{R}\leq K_{H}+2{.75B}_{M}\;\\ k_{R}-2{.75B}_{M}\;\mathrm{otherwise}\; \end{array}$$

A Gaussian-shaped frequency domain window is defined as

(23) $$W_{F}\left[f\right]{=e}^{\frac{{-(f-f}_{0}{)}^{2}}{0.72{B_{F}}^{2}}}\;$$

In Figure 7b, *B*_F_ is the 3 dB wavenumber bandwidth. By multiplying the Tukey and Gaussian windows, the original frequency–wavenumber domain data ${V(k}_{x}{,k}_{y},f)$ were filtered to only one isolated mode, i.e., $\tilde{V}(k_{x}{,k}_{y},f)$.

### 4.2. Local Wavenumber Estimation

The estimation of the local wavenumber for each spatial point, ${\hat{k}}_{\mathrm{LOC}}\left[x,y\right]$, could be achieved by finding the wavenumber bin with the highest magnitude in a space-wavenumber representation by

(24) $${\hat{k}}_{\mathrm{LOC}}[x,y]=\underset{k_{c}}{\mathrm{argmax}}{(z}_{1}{[x,y,k}_{c}])$$

The generation of the space-wavenumber data $z_{1}{[x,y,k}_{c}]$ was as follows. 

The frequency–wavenumber domain signal of one isolated mode, $\tilde{V}(k_{x}{,k}_{y},f)$, was passed through a set of narrowband wavenumber filters parameterized with the central wavenumber *k*_c_, as

(25) $$W_{K}{[k}_{x}{,k}_{y}{,k}_{c}{]=e}^{\frac{-(\sqrt{k_{x}^{2}{+k}_{y}^{2}}{-k}_{c}{)}^{2}}{0{.72B}_{K}^{2}}}\;$$

which is also a Gaussian-shaped window with a 3 dB wavenumber bandwidth $B_{K}$, as shown in Figure 7c. The narrowband wavenumber domain signal was obtained according to

(26) $${Z[k}_{x}{,k}_{y}{,F,k}_{c}]=\frac{\tilde{V}\left[k_{x}{,k}_{y},f\right]W_{K}\left[k_{x}{,k}_{y}{,k}_{c}\right]}{\sum_{k_{x}{,k}_{y}}W_{K}{[k}_{x}{,k}_{y}{,k}_{c}]}$$

The inverse Fourier transform method was applied to the narrowband signal ${Z[k}_{x}{,k}_{y}{,f,k}_{c}]$ to obtain a band-limited space-time-wavenumber representation of the data ${z[x,y,t,k}_{c}]$. This representation was then envelope-summed [23] across time to obtain the desired space–wavenumber representation

(27) $$z_{1}{[x,y,k}_{c}]=\sum_{t}\left|z\left[{x,y,t,k}_{c}\right]\right|$$

Finally, the local wavenumbers were estimated by determining the wavenumber bin with the highest magnitude for each spatial point according to Equation (24).

In summary, the key parameters in the local wavenumber method include:

i. $\hat{K}(f,m)$, the central wavenumber of the mode to be isolated;
ii. *f*_0_, the central frequency of the mode to be isolated;
iii. *C*_L_ and *C*_H_, the lower and upper cutoffs in the Tukey window;
iv. *B*_M_, the half-power taper bandwidth in the Tukey window;
v. *B*_F_, the 3 dB frequency bandwidth in the Gaussian window;
vi. *B*_K_, the 3 dB wavenumber bandwidth in the Gaussian window;
vii. *k*_c_, a bank of possible wavenumber values.

## 5. Experimental Investigations

In this section, a metal-lined COPV cylinder is manufactured with interfacial debonding and impact damage. A laser-generated ultrasonic imaging system is used to scan the CPOV structure. The laser-ultrasonic wavefield is visualized. Dispersion curves are analyzed experimentally and compared with those calculated theoretically to evaluate the interface debonding. In addition, the impact damage is visualized based on the local wavenumber estimation method.

### 5.1. Setup and Wavefields

**Figure 8:**
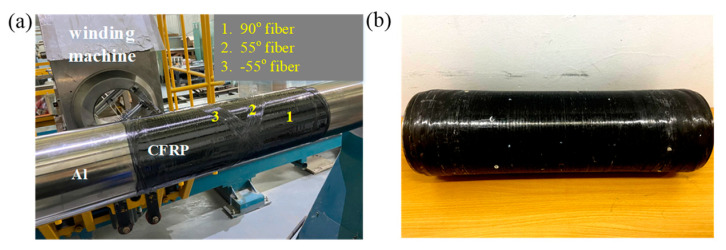
(**a**) The filament-wound COPV during the manufacturing process and (**b**) the COPV cylinder.

Figure 8 shows the COPV cylinder, which was constructed using the same geometry and material as described in Section 3 [32]. To imitate interface debonding, the aluminum liner was wound directly with carbon-fiber bundles that went through the epoxy grooves after the surface was cleaned. The impact damage was induced by a quasi-static indentation test [30,31] through an indenter with a diameter of 12.7 mm and a loading rate of 5 mm/min. After approximately 7 min, the experiment was terminated with a few sounds of fiber breakage. 

**Figure 9:**
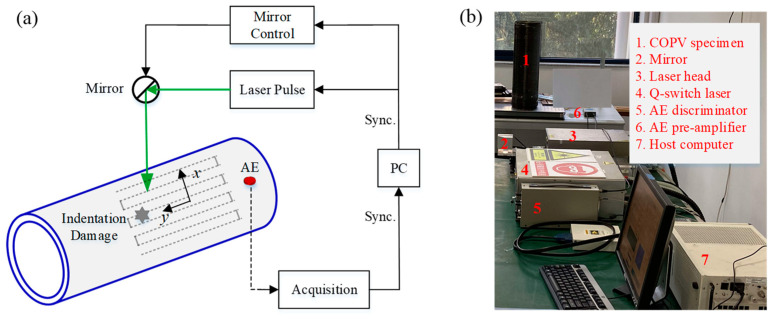
Experimental setup of the laser-generated ultrasonic system. (**a**) schematic diagram and (**b**) photograph.

A laser-generated imaging system was introduced [20,27] for damage detection. As shown in Figure 9, Q-switched laser pulses (an Nd:YLF laser from the Advanced Optowave Corporation Awave series) were used to generate broadband ultrasonic waves, and an acoustic emission sensor (M31, FUJI Ceramics Corporation) pasted on the outer surface served as the receiver. 

The scanning laser-ultrasonic system has numerous advantages, such as its non-contact, remote excitation, high repetition rate and high resolution. The first two advantages are more pronounced in curved structures, such as COPVs. The laser pulse was manipulated to scan the COPV specimen with an area of 100 mm × 200 mm at a spatial interval of 0.5 mm along both scanning axes with real-time mirror control. The scanning repetition rate was 1 kHz, allowing for a rapid scanning process of only 80 s. 

The laser pulse energy was carefully selected to prevent ablation on the surfaces of the COPVs. The time domain signals received by the sensor at each scanning grid were amplified with the AE9922 tool (NF Corporation) using 30 dB at a sampling rate of 5 MS/s. After a full scan, a three-dimensional wavefield data matrix v(x,y,t) can be obtained.

**Figure 10:**
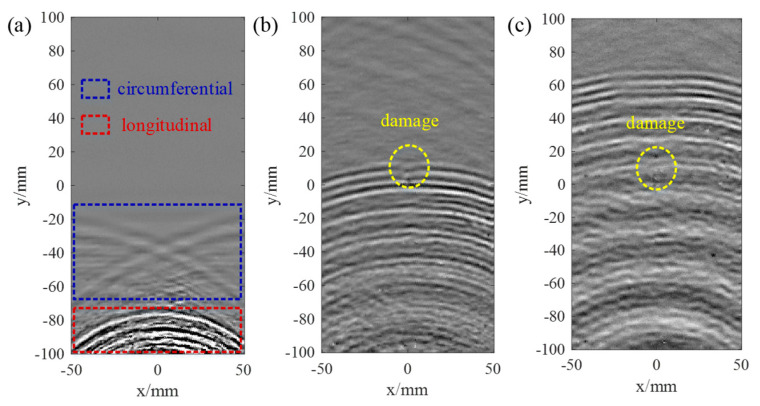
Snapshots of the wavefields at (**a**) 40 μs, (**b**) 100 μs and (**c**) 140 μs.

The wavefield snapshots of the laser-generated guided waves in the COPV are shown in Figure 10. In Figure 10a, both circumferential and longitudinal guided waves are observed. The circumferential modes had relatively higher velocities than the longitudinal modes since the fibers were mostly circumferentially wound. Moreover, as shown in Figure 10b,c, the amplitude of the circumferential modes was smaller than the longitudinal ones. Therefore, for simplicity, the circumferential modes were filtered out in the following study. 

### 5.2. Interface Debonding Evaluation

As previously stated, interfacial debonding can be assessed by studying the dispersion characteristics of the guided waves [33]. 

**Figure 11:**
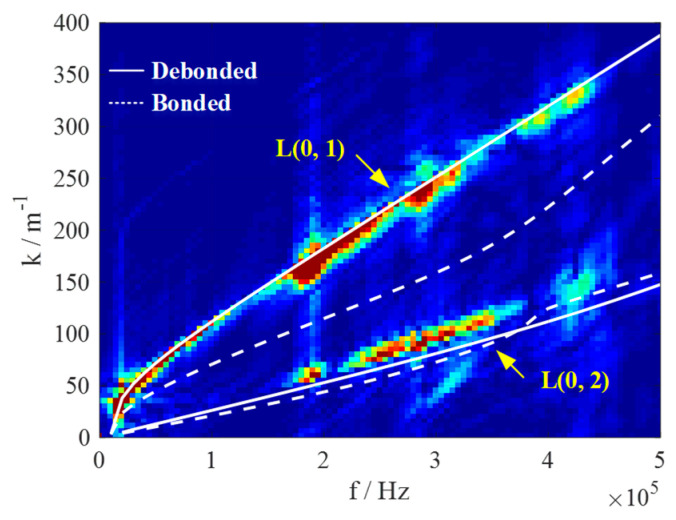
Dispersion curves from experimental measurements and theoretical calculations.

As shown in Figure 10b,c, the two-dimensional time–space signals of 400 scanned points along the axial direction of *x* = 0 mm were converted to the frequency–wavenumber domain using the two-dimensional Fourier transform method [34]. Figure 11 shows the spectrum in the frequency–wavenumber domain, where two guided wave modes could be observed, namely L(0, 1) and L(0, 2).

The theoretical dispersion curves were also plotted. It was found that the theoretical dispersion curves of the COPV with the bonded metal-composite interface were very different from the experimental spectrum. In addition, as expected, the theoretical dispersion curves of the COPV without the aluminum liner matched well with the experimental ones, demonstrating that the structure was debonded at the metal-composite interface. The slight misalignment of the dispersion curves may be attributed to the unavoidable differences between the actual materials and those listed in Table 1, as well as the neglect of the effect of fiber waviness due to the winding process. 

### 5.3. Impact Damage Identification

**Figure 12:**
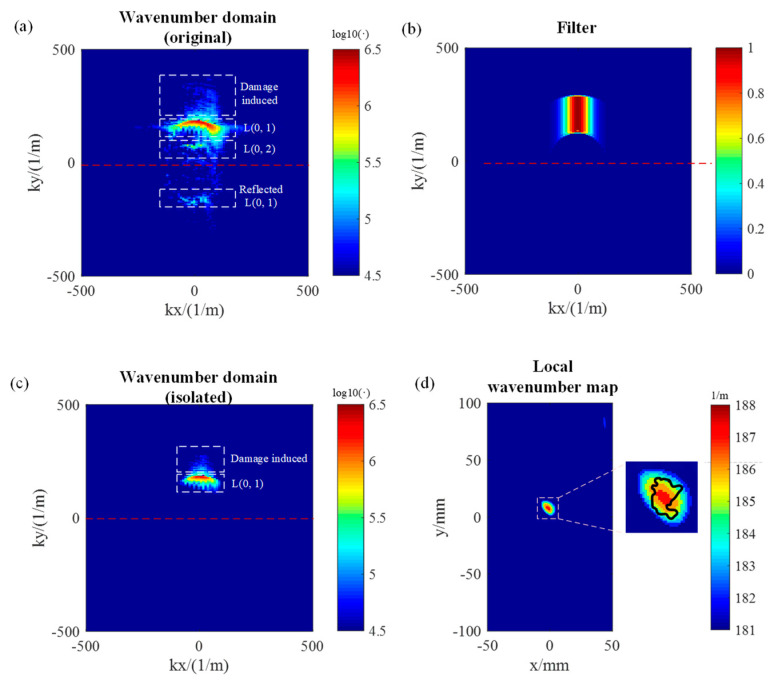
Local wavenumber estimation method. (**a**) wavenumber domain spectrum before filtering, (**b**) filter of the wavenumber domain, (**c**) wavenumber domain spectrum after filtering (**d**) local wavenumber map and C-scan result (black curves).

Based on the theoretical analysis in Section 3, the wavenumber of the L(0, 1) mode was chosen to detect the impact damage. The central frequency was set to 200kHz, where the energy was relatively high according to Figure 11. The wavenumber domain signal is shown in Figure 12a, where the L(0, 2) mode needed to be removed. The reflected L(0, 1) mode was less intense compared to the emitted L(0, 1) mode and could also be neglected. 

In the local wavenumber estimation method, the main parameters were set as follows.

i. For the Tukey window to filter the wavenumber domain, ${\hat{K}(f,m)=180\;m}^{-1}$, $C_{L}{=50\;m}^{-1}$, $C_{H}{=100\;m}^{-1}$, $B_{M}{=4\;m}^{-1}$;
ii. For the Gaussian window to filter the frequency domain, $f_{0}=200\;\mathrm{kHz}$, $B_{F}=20\;\mathrm{kHz}$;
iii. For the two-dimensional Gaussian window to filter the possible local wavenumber, $k_{c}{=[160,\;220]\;m}^{-1}$ with an interval of $0{.2\;m}^{-1}$, $B_{K}{=30\;m}^{-1}$.

In addition to the Tukey window, an additional treatment was applied to the wavenumber domain signal to eliminate the reflected L(0, 1) modes by setting the intensity value of the negative wavenumber to zero. To reduce the effect of the wave propagating beyond the axial direction, another Gaussian window was applied to the wavenumber domain with a central wavenumber of $k_{x}{=0\;m}^{-1}$ and a 3 dB wavenumber bandwidth of ${35\;m}^{-1}$. The final filter of the wavenumber domain is shown in Figure 12b. The filtered spectrum of the wavenumber domain is shown in Figure 12c, where only the focused L(0, 1) mode and the damage-induced L(0, 1) mode were present. The local wavenumber map is shown in Figure 12d, and the color map was re-edited to highlight the damage region, while the true wavenumbers of the healthy and damaged regions were approximately 174 m^−1^ and 187 m^−1^, respectively.

The local wavenumber estimation method carries more information because it is closely related to structural or material properties [23,26]. The impact damage was approximately 12 mm × 10 mm according to Figure 12d, and the depth was approximately 0.4 mm based on Figure 5.

To verify the detected depth of the impact damage, a depth gauge with an accuracy of 0.01 mm (F1402, Qinghai Gauge & Sharpening Co., Ltd., Xining, China) was used to measure the depth of the crater. The average value of the multiple measurements was approximately 0.38 mm, which was very close to the result estimated with the local wavenumber method.

The conventional C-scan method was utilized to verify the in-plane size of the impact damage. An ultrasonic probe with a central frequency of 5 MHz was used to generate and receive ultrasonic pulses with a sampling frequency of 100 MHz. A two-dimensional spatial drive motor system was established to accomplish the in-plane motion of the transducer. The amplitude of the first ultrasonic peak reflected from the COPV surface was collected to form the C-scan image. The boundary of the impact damage in the C-scan result was provided as black curves in Figure 12d. It was shown that the location of the impact damage is detected correctly by the local wavenumber estimation method. As mentioned above, the damage size estimated with the local wavenumber method was approximately 12 mm × 10 mm, while the C-scan result was approximately 10 mm × 8 mm. The size of the impact damage detected with the local wavenumber method was slightly larger than that detected with the C-scan method due to the use of the Gaussian window in Equation (25) during signal processing, which inevitably increases the image size.

## 6. Conclusions

This paper investigated the ultrasonic-guided wave method for the non-destructive evaluation of a filament-wound COPV with a metal liner. An eigenfrequency method was utilized for the analysis of the dispersion characteristics of the COPV without the need for additional coding. A laser-ultrasonic system was used to rapidly scan the COPV to detect the interfacial debonding and impact damage. The conclusions drawn are as follows.

i. In the eigenfrequency approach, a specific unit cell of the COPV with a Floquet BC was demonstrated as feasible for analyzing the dispersion characteristics. The accuracy of the method was validated with the SAFE method. In fact, the eigenfrequency method is applicable to any type of waveguide, including pipes, plates, etc., and to both isotropic and anisotropic materials.

ii. Based on the eigenfrequency method, the effects of the interfacial debonding and impact damage on the wave characteristics were investigated. The dispersion curves were suitable for the assessment of metal-composite interface debonding, and the wavenumber of the L(0, 1) mode was selected for impact damage detection.

iii. The laser-ultrasonic wavefield contained both circumferentially and longitudinally propagating waves and exhibited multi-mode characteristics. By comparing the experimental dispersion curves with the theoretical ones, it was demonstrated that the metal and composite materials were not bonded to the metal–CFRP interface. Quantitative studies regarding the interfacial debonding state need to be carried out in the future.

iv. The impact damage was visualized with the local wavenumber estimation method, and the wavenumber of the L(0, 1) mode in the healthy and damaged regions were approximately 174 m^−1^ and 187 m^−1^, respectively. Based on the local wavenumber map, the size and depth of the impact damage were roughly estimated. The C-scan method was used to verify that the in-plane size and depth were close to that measured using the depth gauge.

## Figures and Tables

**Table 1:** Material properties of the COPV.

Properties of liner (Al)	*E* = 70 GPa, *ν* = 0.32, *ρ* = 2700 kg/m^3^
Properties of single layer in the composite (CFRP)	*E*_1_ = 158 GPa, *E*_2_ = *E*_3_ = 3 GPa, *ν*_12_ = *ν*_13_ = 0.307, *ν*_23_ = 0.45, *G*_12_ = *G*_13_ = 4.71 GPa, *G*_23_ = 3.99 GPa; *ρ* = 1600 kg/m^3^

## Data Availability

Not applicable.

## References

[1] Rajak D.K., Pagar D.D., Kumar R., Pruncu C.I. (2019). Recent progress of reinforcement materials: A comprehensive overview of composite materials. J. Mater. Res. Technol..

[2] Wang D., Liao B., Zheng J., Huang G., Hua Z., Gu C., Xu P. (2019). Development of regulations, codes and standards on composite tanks for on-board gaseous hydrogen storage. Int. J. Hydrogen Energy.

[3] Barthélémy H., Weber M., Barbier F. (2017). Hydrogen storage: Recent improvements and industrial perspectives. Int. J. Hydrogen Energy.

[4] Sun H., Kosukegawa H., Hashimoto M., Uchimoto T., Takagi T. (2020). Electromagnetic-pulse-induced acoustic testing for nondestructive testing of plastic composite/metal adhesive bonding. Int. J. Hydrogen Energy.

[5] Wang L., Wang B., Wei S., Hong Y., Zheng C. (2016). Prediction of long-term fatigue life of CFRP composite hydrogen storage vessel based on micromechanics of failure. Compos. Part B Eng..

[6] Weerts R.A., Cousigné O., Kunze K., Geers M.G., Remmers J.J. (2021). The initiation and progression of damage in composite overwrapped pressure vessels subjected to contact loads. J. Reinf. Plast. Compos..

[7] Long B., Yang N., Cao X. (2022). Low-velocity impact damages of filament-wound composite overwrapped pressure vessel (COPV). J. Eng. Fibers Fabr..

[8] Weerts R.A., Cousigne O., Kunze K., Geers M.G., Remmers J.J. (2021). A methodological approach to model composite overwrapped pressure vessels under impact conditions. Compos. Struct..

[9] Zhang M., Lv H., Kang H., Zhou W., Zhang C. (2019). A literature review of failure prediction and analysis methods for composite high-pressure hydrogen storage tanks. Int. J. Hydrogen Energy.

[10] Wu L., Wang W., Jiang Q., Xiang C., Lou C.W. (2019). Mechanical characterization and impact damage assessment of hybrid three-dimensional five-directional composites. Polymers.

[11] Elkolali M., Nogueira L.P., Rønning P.O., Alcocer A. (2022). Void Content Determination of Carbon Fiber Reinforced Polymers: A Comparison between Destructive and Non-Destructive Methods. Polymers.

[12] Kim T., Deveci S., Yang I., Stakenborghs B., Choi S. (2022). Visual, Non-Destructive, and Destructive Investigations of Polyethylene Pipes with Inhomogeneous Carbon Black Distribution for Assessing Degradation of Structural Integrity. Polymers.

[13] Rose J.L. (2014). Ultrasonic guided waves in solid media.

[14] Silk M., Bainton K. (1979). The propagation in metal tubing of ultrasonic wave modes equivalent to Lamb waves. Ultrasonics.

[15] Thomson W.T. (1950). Transmission of elastic waves through a stratified solid medium. J. Appl. Phys..

[16] Knopoff L. (1964). A matrix method for elastic wave problems. Bull. Seismol. Soc. Am..

[17] Yang Z., Wu Z. (2020). Guided Waves Dispersion Analysis in Composite Pipe Using the SAFE Method. Proceedings of the European Workshop on Structural Health Monitoring.

[18] Hakoda C., Rose J., Shokouhi P., Lissenden C. (2018). Using Floquet periodicity to easily calculate dispersion curves and wave structures of homogeneous waveguides. AIP Conf. Proc..

[19] Lee J.H., Lee S.J. (2009). Application of laser-generated guided wave for evaluation of corrosion in carbon steel pipe. Ndt E Int..

[20] Wang H., Zhang C., Ji H., Qiu J. (2022). Damage visualization using laser-generated residual guided waves with optimization of laser scanning path. Mech. Syst. Signal Processing.

[21] An Y.K., Park B., Sohn H. (2013). Complete noncontact laser ultrasonic imaging for automated crack visualization in a plate. Smart Mater. Struct..

[22] Tian Z., Yu L., Leckey C., Seebo J. (2015). Guided wave imaging for detection and evaluation of impact-induced delamination in composites. Smart Mater. Struct..

[23] Flynn E.B., Chong S.Y., Jarmer G.J., Lee J.R. (2013). Structural imaging through local wavenumber estimation of guided waves. Ndt E Int..

[24] Segers J., Hedayatrasa S., Poelman G., Van Paepegem W., Kersemans M. (2022). Self-reference broadband local wavenumber estimation (SRB-LWE) for defect assessment in composites. Mech. Syst. Signal Processing.

[25] Tian Z., Xiao W., Ma Z., Yu L. (2021). Dispersion curve regression–assisted wideband local wavenumber analysis for characterizing three-dimensional (3D) profile of hidden corrosion damage. Mech. Syst. Signal Processing.

[26] Lugovtsova Y., Bulling J., Mesnil O., Prager J., Gohlke D., Boller C. (2021). Damage quantification in an aluminium-CFRP composite structure using guided wave wavenumber mapping: Comparison of instantaneous and local wavenumber analyses. NDT E Int..

[27] Zhao J., Qiu J., Ji H. (2016). Reconstruction of the nine stiffness coefficients of composites using a laser generation based imaging method. Compos. Sci. Technol..

[28] Ashcroft N.W., Mermin N.D. (1976). Solid State Physics.

[29] (2015). COMSOL Multiphysics: Reference Manual.

[30] Wang R., Chen P., Shen Z. (2008). Damage resistance analysis of composite laminates subjected to quasi-static indentation. Acta Mater. Compos. Sin..

[31] Sun C., Potti S. (1996). A simple model to predict residual velocities of thick composite laminates subjected to high velocity impact. Int. J. Impact Eng..

[32] Gu F., Gu Z., Zhu X., Lu X., Fang D., Li L. (2021). Design and hydraulic tests of a metal liner composite overwrapped pressure vessels with seamless connection technology. Acta Mater. Compos. Sin..

[33] Zabbal P., Ribay G., Chapuis B., Jumel J. (2018). Multichannel Multiple Signal Classification for dispersion curves extraction of ultrasonic guided waves. J. Acoust. Soc. Am..

[34] Alleyne D. (1991). A two-dimensional Fourier transform method for the measurement of propagating multimode signals. J. Acoust. Soc. Am..

